# Understanding the Role of Cognitive Abilities and Math Anxiety in Adolescent Math Achievement

**DOI:** 10.3390/jintelligence13040044

**Published:** 2025-04-03

**Authors:** Lorenzo Esposito, Irene Tonizzi, Maria Carmen Usai, David Giofrè

**Affiliations:** DISFOR, University of Genoa, Corso Andrea Podestá, 2, 16121 Genova, Italy; irene.tonizzi@edu.unige.it (I.T.); maria.carmen.usai@unige.it (M.C.U.)

**Keywords:** math, higher-order cognitive abilities, working memory, inhibitory control, executive functions, math anxiety, children

## Abstract

A consistent amount of research has tried to study the contributions of cognitive and emotional factors involved in math achievement. Despite this, research examining their joint role in children is scarce. In this paper, we examined the joint role of cognitive and math anxiety on math achievement in a sample of 135 seventh-grade children (54% male, *M_age_* = 12.79, *SD* = 0.47). Math achievement was measured using a validated paper-and-pencil test, while higher-order cognitive abilities were assessed with a PMAs test. Working memory was evaluated through two verbal and two visuo-spatial experimental span tasks. Inhibitory control was measured using three computerized tasks adapted from the classic Stroop, Flanker, and Simon tasks. Math anxiety was assessed with an AMAS questionnaire. A series of correlation analyses and path models were conducted to understand the complex relationships among the factors. The correlations showed a positive relationship among our cognitive abilities and a negative correlation with math anxiety. The results from the path analysis showed a strong effect of higher-order cognitive abilities on math achievement (β = 0.44, *p* < .001) and highlighted the mediating role of working memory between math anxiety and math performance (β = −0.04, 95%CI [−0.11; −0.00]). Conversely, inhibitory control did not seem to play a crucial role in this relationship (β = −0.03, 95%CI [−0.08; 0.00]). These findings are discussed in relation to current theoretical frameworks. Interventions aimed at reducing math anxiety could help improve math achievement.

## 1. Introduction

Learning to count, acquiring number skills, performing arithmetical operations, and solving complex math problems constitute an important part of children’s daily activities and remain important throughout adulthood. Research on mechanisms underlying math learning has a long history, and according to the most recent developmental theories, the acquisition of mathematical abilities depends on both domain-specific and domain-general factors (Caviola et al. 2020; Hornung et al. 2014; Passolunghi and Lanfranchi 2012). Among the math-specific factors, we can list several skills, such as comparing sets of objects and counting abilities, preceding the acquisition of numerals knowledge (Krajewski and Schneider 2009; Purpura et al. 2013). Also, math, like any other complex achievement, relies on higher cognitive abilities, such as intelligence (Giofrè et al. 2013a, 2017) and reasoning (H. L. Johnson 2015; Nunes et al. 2015). Among those, working memory (Caviola et al. 2014; Giofrè et al. 2014) and executive functions (Borella and de Ribaupierre 2014; Cragg and Gilmore 2014; Simanowski and Krajewski 2019; Usai et al. 2018; Viterbori et al. 2017) have consistently been associated with math. Emotional factors seem to play a role in math as well, and one of the most significant is math anxiety (Ashcraft and Moore 2009; Caviola et al. 2022; Donolato et al. 2020).

### 1.1. Higher-Order Cognitive Abilities

According to several studies, academic achievement is related to some cognitive functions, such as verbal, non-verbal, and quantitative abilities, that are used as indicators of higher-level cognitive functions (Demetriou et al. 2013; W. Johnson et al. 2008). Consistently, several studies that analyzed international data found a strong relationship between academic achievement and higher-order abilities, such as abstract reasoning (Frey and Detterman 2004; Pokropek et al. 2022). Recent research has suggested that these higher-level cognitive functions might mediate the relationship between lower-order cognitive abilities (e.g., working memory) and math performance, such as general math achievement (Giofrè et al. 2017) and math reasoning (Qi et al. 2024). According to this framework, lower-level functions (e.g., working memory, inhibitory control) are integrated into higher-level intellective functions (e.g., fluid intelligence, analogical reasoning) in a cascading fashion during development (Demetriou et al. 2013, 2014; Giofrè et al. 2017; Qi et al. 2024). In other words, the age-related development of lower-order abilities is necessary for the development of higher-order abilities (Demetriou and Spanoudis 2017; Tourva and Spanoudis 2020).

### 1.2. Executive Functions

Executive functions (EFs) appear to be particularly important for math learning (Purpura et al. 2017; Simanowski and Krajewski 2019). EFs are a set of cognitive processes that support goal-directed behavior and are involved in planning, controlling, and supervising cognition and behavior, especially in new or cognitively demanding situations (Miyake et al. 2000; Miyake and Friedman 2012). According to Miyake’s model, there are three separated, but interrelated, functions: inhibitory control, updating, and shifting. EFs develop over time and seem to be a relatively undifferentiated construct in young children (Wiebe et al. 2010). EFs start to differentiate from 5 to 7 years of age (Lerner and Lonigan 2014; M. R. Miller et al. 2012; Usai et al. 2014) and reach the typical adult three-component structure starting from 8 to 13 years of age (Lee et al. 2013). The relationship between EFs and math is well documented (Cragg et al. 2017; Cragg and Gilmore 2014; Emslander and Scherer 2022; Viterbori et al. 2017). EFs are involved in complex cognitive tasks such as arithmetic and algebraic reasoning (Agostino et al. 2010; Andersson 2007; Lee et al. 2004), calculations (Andersson 2008; Berg 2008), early numeracy (Bull and Scerif 2001; Campos et al. 2013), and general math achievement (Kroesbergen et al. 2009; Navarro et al. 2011). 

However, multiple studies have found no correlation between shifting abilities and academic achievement. For example, no relationship between shifting abilities and performance on math achievement exams in adolescents has been consistently found (Cragg et al. 2017; Friso-Van Den Bos et al. 2013). It is plausible that younger children may rely more on these abilities, but their importance decreases as procedural skills become more automatic with age (Cragg and Gilmore 2014). Critically, two meta-analyses have shown a weak association between shifting abilities and math achievement (Jacob and Parkinson 2015; Yeniad et al. 2013). It is worth noting that discrepancies in the literature can be attributed to several factors, including differences in age groups and heterogeneity among tasks that assess shifting abilities (Li et al. 2020). These factors highlight the complexity of linking shifting abilities to math performance and suggest that its impact might be dependent on context.

Inhibitory control (IC) seems to be particularly important in math learning due to its specific role in suppressing irrelevant or interfering information. Math learning is not just about acquiring new information; it also requires updating existing conceptual frameworks and forming new concepts (Rochat 2023). IC may influence math achievement directly or by aiding other cognitive abilities (e.g., working memory) by discarding irrelevant information, thus leading to better performances (Bull and Lee 2014; Lee and Lee 2019; Miyake et al. 2000). Specifically, there is evidence that IC is related to math, in which it is necessary to prevent prior knowledge, intuitive ideas, and even erroneous perceptual cues from interfering with learning paradoxical notions (Stavy and Tirosh 2000). A classic example of this phenomenon is in fraction comparison, where children tend to be slower and less accurate in selecting the larger fraction when the correct answer conflicts with their knowledge of whole numbers (e.g., 1/3 is larger than 1/10, but 3 is smaller than 10) (Bonato et al. 2007). In this framework, several studies suggest that children and adolescents with better IC perform better on apparently paradoxical and counterintuitive math problems (Borst et al. 2013; Brookman-Byrne et al. 2018; Lubin et al. 2013; Wilkinson et al. 2020).

### 1.3. Working Memory

Working memory (WM) refers to a processing resource of limited capacity that is involved in the temporaneous storing of information while simultaneously processing the same or other information (Baddeley 2000). WM is involved in complex tasks, such as arithmetical problem-solving, mental calculations (Chen and Bailey 2021; Zhang et al. 2023), symbolic and non-symbolic comparison (Caviola et al. 2020; Nelwan et al. 2022; van Dijck and Fias 2011), geometrical problem-solving (Giofrè et al. 2013b, 2014; Rivella et al. 2021), and general math achievement (Friso-Van Den Bos et al. 2013). Many studies have used the multi-componential WM model, initially developed by Baddeley and Hitch (Baddeley 2000), as the preferred framework when studying the role of WM and its relations with math. A recent meta-analysis found that the overall association between WM and math is *r* = 0.35, and is moderated by the specific math skill analyzed (e.g., computation *r* = 0.35, geometry *r* = 0.23; Peng et al. 2016).

### 1.4. Math Anxiety

In addition to cognitive factors, such as WM and EFs, other factors were found to be linked to math (e.g., emotional aspects; Donolato et al. 2020). Extensive research has suggested that academic anxiety, a type of anxiety experienced in educational settings, negatively affects performance across a variety of school subjects (Caviola et al. 2022; Zeidner 2007). In particular, math anxiety (MA) is considered a specific form of academic anxiety, not limited to testing situations but extended to any moment a student may deal with math content (Ashcraft and Moore 2009; H. Miller and Bichsel 2004). MA can be defined as a feeling of fear, nervousness, or discomfort that interferes with one’s ability to perform math tasks, thus having a detrimental effect on students’ math achievement (Ashcraft 2002). Research has shown that test anxiety and MA may negatively affect math achievement indirectly through WM resources, although this effect is negligible in magnitude (Caviola et al. 2022). However, it is worth noting that MA may directly affect math achievement as well, however, the reasons seem to be unclear. It is plausible that higher levels of MA, extended throughout school years, can eventually trigger avoidance behavior (Choe et al. 2019). In fact, people with high MA might be afraid of performing math tasks, so they often avoid math-oriented university courses (Daker et al. 2021).

### 1.5. Aim and Hypotheses

As outlined above, a consistent amount of research has tried to study the contributions of domain-specific and domain-general cognitive and emotional factors involved in math. The main aim of the present study was to examine the joint role of cognitive (e.g., higher-order cognitive abilities, WM, and IC) and emotional (e.g., MA) factors in math among seventh graders. There is, in fact, a growing amount of research looking at the effects of both cognitive (Giofrè et al. 2014, 2017; Roth et al. 2015) and emotional factors (Beilock and Maloney 2015; Foley et al. 2017) on math achievement. The present study aims to determine the extent to which WM and IC contribute to math achievement and to investigate whether higher-order cognitive abilities mediate the relationship between these cognitive functions and math performance. In this study, we refer to higher-order cognitive abilities (HCA) as the set of core cognitive abilities involved in math performance. Also, while previous research has primarily focused on the interfering role of MA in cognitive functions (i.e., WM), recent evidence suggests that MA may also have a direct negative impact on math performance (Caviola et al. 2022). Additionally, HCA are expected to play a key role in shaping the relationship between cognitive abilities and math achievement, influencing how WM and IC contribute to performance. Based on prior research, we hypothesize (1) that better performance in WM and IC is positively correlated with better math achievement. These factors are expected to play a critical role, with WM supporting math and IC aiding problem-solving and cognitive control during these tasks. As outlined above, research has suggested a potential effect of HCA on the relationship between cognitive functions and math. Therefore, we expect the HCA to predict math achievement above WM and IC (2), and to mediate the effects of WM and IC on math achievement (2a). Higher levels of MA are expected to negatively affect performance in math, leading students with higher levels of MA to show worse results in math (3). Crucially, there is an indirect effect through its interference with WM and IC (3a), as well as a direct effect of MA on math achievement (3b).

## 2. Materials and Methods

### 2.1. Participants

The sample consisted of seventh-grade children recruited from three schools in Genova (Italy). Forty participants with intellectual disabilities, specifically learning disorders, including educational diseases, neurological disorders, and genetic syndromes, were excluded. Seven participants were also excluded because they were found to be multivariate outliers and possible influential cases, based on Cook’s distance. The final sample was composed of 135 participants (73 males and 62 females), with an age range in years of 11.92–15.10; *M* = 12.79, *SD* = 0.47 (in months 143.129–181.29; *M* = 153.49; *SD* = 5.58). This study was approved by the Ethical Committee of the University of Genova (protocol code n. 2024.13, 20 February 2024).

### 2.2. Design and Procedure

All the children were tested individually in a quiet room at their school. The tests included three computerized tasks for IC, four computerized tasks for WM (two for verbal WM and two for visuo-spatial WM), two paper-and-pencil tests to assess HCA and math, and a questionnaire to measure MA. The seven computerized tasks were administered in a pseudo-randomized order (Stroop Task, Flanker Task, Simon Task, dual matrices task, back verbal task, dual verbal task, and back matrices task) to reduce the effects of fatigue and practice. Due to time constraints, tasks were administered in separate sections: (i) the computerized tasks, which lasted about 1 h, (ii) the HCA test, alongside the MA questionnaire, which lasted about 30 min, and (iii) the math test, which lasted about 1 h.

### 2.3. Measures

The IC measures were adapted from the literature for the child population (Burgoyne et al. 2023). Two instruments, which were already validated in the Italian sample, were used to assess math achievement (Amoretti et al. 1997) and MA (Caviola et al. 2017). The WM measures were previously used in other studies with similar populations (Allen et al. 2020; Giofrè et al. 2017). 

#### 2.3.1. Math

Math was assessed with a math test for basic education (Amoretti et al. 1997). It is a standardized test that provides a year group-specific measure of math achievement, in line with the objectives of the national curriculum. Children are required to develop an understanding of numeracy, geometry, and basic statistics, according to the national curriculum in Italy. The score considered in this test was computed as the sum of the correct responses (Cronbach’s alpha, *α* = 0.70).

#### 2.3.2. Higher-Order Cognitive Abilities

The Primary Mental Abilities (PMAs) tests assess different cognitive abilities (Thurstone 1937). The scores considered in these subtests were computed as the sum of correct responses. As proposed by W. Johnson et al. (2008), several cognitive tests, despite focusing on individual abilities, were shown to be closely correlated, indicating that they measure the general intelligence factor. The three cognitive abilities tested in this study, spatial, verbal, and reasoning abilities, are important parts of cognition and have been demonstrated to be strongly related to general intelligence (W. Johnson et al. 2008; W. Johnson and Bouchard 2005). The scores obtained in these tests were used as a measure of HCA.

##### Spatial Ability Test (PMA-S)

This test required the children to identify identical figures among six rotated figures, some of which were also mirrored. This task consisted of 20 items to be completed within 5 min, each offering multiple correct options (*α* = 0.86).

##### Verbal Ability Test (PMA-V)

This test involved selecting synonyms for given words among four options, such as choosing “to look” as a synonym for “to watch”. This paper-and-pencil test included 50 items to be completed within 4 min (α = 0.80).

##### Reasoning Ability Test (PMA-R)

This test required completing letter sequences by selecting the logically correct letter, for instance, identifying “h” as the next letter in sequences like “aab”, “ccd”, “eef”, and “gg”. This test included 30 items to be completed within 6 min (α = 0.85). 

#### 2.3.3. Inhibitory Control

The three measures of IC were taken using the Stroop Task, Flanker Task, and Simon Task. Importantly, these tasks have already been used on adult participants (Burgoyne et al. 2023), so an adaptation was needed for the child population. Specifically, unlike the original ones, a cut-off of 2 minimum correct responses in the training block was added to ensure that participants understood the instructions to perform in the test block. In addition, the feedback was given only at the end of the training block instead of after each trial. These tasks were designed to add an additional level of conflict to each of the traditional conflict paradigms at the response level. The scores considered for these tasks were computed by the sum of incorrect responses subtracted from the sum of correct responses. 

##### Stroop Task

In this task, children are shown a target stimulus in the center of the screen with two response options below it. The task follows the typical Stroop paradigm, where a response must be made to the display color and not the semantic meaning of the target stimulus (“RED” or “BLUE” displayed in red or blue colors). The task challenges participants to focus on the display color of the target stimulus and the semantic meaning of the response options. At the same time, they must ignore the semantic meaning of the target stimulus and the display color of the response options. For example, if the target stimulus is the word “RED” appearing with a blue display color, the child must select the response option that says the word “BLUE,” regardless of the response option display color (*α* = 0.96).

##### Flanker Task

In this task, children are presented with a target stimulus and two response options. Both the target stimulus and response options consist of flanker items made up of five arrows (e.g., > > < > >). The task requires participants to focus on the flanking arrows of the target stimulus and the central arrow of the response options. In contrast, they must ignore the central arrow of the target stimulus and the flanking arrows of the response options. For example, given the following target stimulus (e.g., < < > < <), the child must select the response option with a central arrow pointing to the left (e.g., > > < > >) (α = 0.94).

##### Simon Task

In this task, children are shown a target stimulus in the form of an arrow, along with two response options: “RIGHT” and “LEFT”. The task is for participants to choose the response option that corresponds to the direction in which the arrow is pointing. The challenge consists of focusing on the direction of the target stimulus arrow and the meaning of the response options. Simultaneously, participants must ignore the side of the screen where the target stimulus arrow and response options appear. For example, if the target stimulus is an arrow pointing left, the child must select the response option that says the word “LEFT.” Complicating matters, the target stimulus arrow and response options can appear on either side of the computer screen with equal probability (α = 0.99).

#### 2.3.4. Working Memory

Four measures of WM were taken: two for verbal WM and two for visuo-spatial WM. These tasks were derived from the previous literature (Giofrè et al. 2017). The total of correct responses is considered a reliable proxy of WM capacity. 

##### Verbal WM

Two measures of verbal WM were considered: backward word span and a verbal dual task. The backward word span task required the children to repeat the list of words they had heard in backward order (*α* = 0.73). The lists of words had lengths spanning gradually from 2 to 8 words, and each span included two trials (70 words in total). The dual task required the children to listen to several word lists, all of length 4. The number of lists spanned gradually from 2 to 6 lists, and each span included two trials (160 words presented in total, 40 words to recall). The children were required to press the spacebar when they heard the name of an animal, as well as retain the last word of each list. The word lists used did not contain any mathematical or geometrical terms, such as “square” or “addition”. Once they had heard all the lists for that trial, the children were asked to recall the last word from each list in the correct order (*α* = 0.68). Both tasks presented words at a rate of one word every 2 s. Before the test phase, each participant practiced an example with a span of two. A score of 1 was assigned for each word recalled correctly, while a score was 0 in cases of incorrect response The final score considered in each of these tasks was computed as the sum of correct responses.

##### Visuo-Spatial WM

Two measures of visuo-spatial WM were taken: backward matrices and a visuo-spatial dual tasks, both using a 4 × 4 grid. The backward (α = 0.71) matrices required the children to repeat the sequence of black squares they had seen in backward order. The sequence of squares had lengths spanning gradually from 2 to 8 squares, and each span included two trials (70 squares in total). The dual task presented a series of grids with several squares colored grey. In each grid, the children saw three black dots sequentially. The children were required to press the spacebar if they saw a dot in a grey square, as well as remember the position of the last (3rd) dot in each grid. Once they had seen all the grids for that trial, the children were asked to recall the positions of the last dots in the correct order (α = 0.75). The number of grids spanned gradually from 2 to 6 grids, and each span included two trials (120 dots presented in total, 40 dots to recall). Both tasks presented stimuli at a rate of one dot/square per 2 s. Before the test phase, each participant practiced an example with a span of two. A score of 1 was assigned for each word recalled correctly, while a score 0 in cases of incorrect response. The final score considered in each of these tasks was computed as the sum of the correct responses.

#### 2.3.5. Math Anxiety

MA was assessed using the Italian standardized version of the Abbreviated Mathematics Anxiety Scale for children (Caviola et al. 2017). This is a self-reported questionnaire composed of 9 items related to learning new math content and the fear of being tested in math. The participants were asked to indicate how they felt about math situations on a scale from 1 to 5. The total score is the sum of all items; a high score indicates a higher level of anxiety toward math (*α* = 0.80).

### 2.4. Data Analysis

Analyses were performed in R (R Core Team 2024), using RStudio (version 2024.12) as the IDE (RStudio Team 2024). The “lavaan” package was used for path analyses (Rosseel 2012). A series of correlations was performed to study the relationships among our variables. We fitted a measurement model to check that the observed variables adequately reflected the latent constructs, confirming the measures’ reliability. As for MA, measured by only one indicator, its variance was fixed using the formula “1-Reliability” (Kline 2011). A series of Confirmatory Factor Analyses (CFAs) was performed to determine the factor structure of each measure. Subsequently, composite scores for each measure were computed as the mean of the raw scores using the regression method, which relies on factor loadings to weight observed variables (Distefano et al. 2008). After having established the factorial structure of WM, IC, HCA, and math achievement, a path modelling approach was used to explore the relationships among WM, IC, HCA, MA, and math achievement.

The goodness-of-fit criteria were evaluated according to guidelines proposed by Hu and Bentler (1999), who suggested a *CFI* (Comparative Fit Index) and *TLI* (Tucker Lewis Index) greater than 0.95 as a good fit, an *RMSEA* (Root Mean Square Error of Approximation) lesser than 0.06 as an acceptable fit, and an *SRMR* (Standardized Root Mean Square Residual) lesser than 0.08 as a good fit (Hu and Bentler 1999). Since model comparison is not possible when models are not nested, the relative index *AIC* (Akaike Information Criterion) was used, where a decrease of 2–4 units was interpreted as an improvement of the model (Burnham and Anderson 2004). Indirect effects were estimated via a Monte Carlo simulation at a significance level of *α* = 0.05, using the “semTools” package (Jorgensen et al. 2022). The Monte Carlo simulation can be used to examine the robustness of estimates and it is particularly useful in complex models (Enders 2020; Paxton et al. 2001).

## 3. Results

### 3.1. Preliminary Analyses

The measurement model (Model 0) was tested via CFAs to study the measures’ reliability. The WM factor was estimated from the four WM tasks, the IC factor from the three IC tasks, the HCA from the three PMAs tests, math from the three subtests, and MA from its variance. Loadings on their respective factors were all statistically significant and ranged from moderate to strong (minimum standardized loading β = 0.44). The results showed a general positive relation between the factors, except for MA, which was negatively related to the other factors. Subsequently, the composite scores were computed for each variable, and to test the hypotheses (1) and (3), we performed a series of correlations to understand the relationships among the variables (see Table 1). The results showed a positive correlation between math achievement and both WM and IC, while a negative correlation between MA and math achievement emerged. Additional correlations were included in the Supplementary Materials (Table S1). Fit indices for Model 0 were all acceptable: *CFI* = 0.97, *TLI* = 0.96, *RMSEA* = 0.04, *SRMR* = 0.07, *AIC* = 6240.47 (see Table 2).

### 3.2. Path Analysis

We tested a series of path models to understand how and to what extent all variables interact with each other. In Model 1, following Hypothesis 2, only WM, IC, HCA, and math were included. In this model, WM, IC, and HCA were all hierarchically at the same level and assumed to predict performance in math (Alloway and Alloway 2010). Not surprisingly, only the HCA predicted math achievement. The fit measures for Model 1 indicated a perfect fit, *CFI* = 1, *TLI* = 1, *RMSEA* < 0.001, *SRMR* < 0.001, and *AIC* = 2073.19. Model 1 is a “just-identified” model whose *df* are equal to 0.

In Model 2, following Hypothesis 2a, we assumed the covariance between WM and IC, and the effect of WM and IC to be fully mediated by the HCA. In this model, WM significantly predicted the HCA, which in turn predicted math achievement. Conversely, IC was not associated with HCA. The fit measures for Model 2 were slightly lower than the previous models, with *CFI* = 0.97, *TLI* = 0.91, *RMSEA* = 0.11, *SRMR* = 0.06, and *AIC* = 2073.66, showing a slight improvement compared to Model 1 (Δ*AIC* = −0.47). The indirect effect of WM on math through HCA was significant, with a coefficient of β = 0.23, 95%CI [0.11; 0.37].

**Figure 1:**
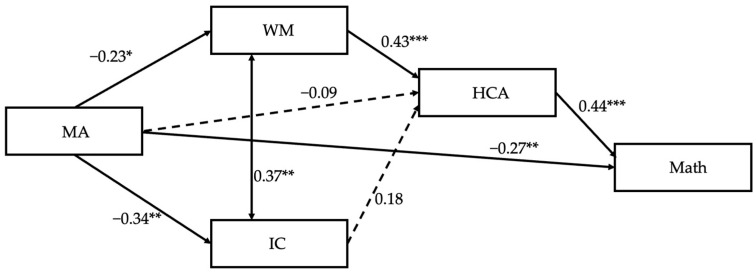
Standardized solution for Model 3. Note. * *p* < .05, ** *p* < .01, and *** *p* < .001.

In Model 3, we added the MA path on all the other variables. The fit measures were *CFI* = 1, *TLI* = 0.99, *RMSEA* = 0.04, *SRMR* = 0.03, and *AIC* = 2064.62, showing an improvement compared to Model 2 (Δ*AIC* = −9.04). Regarding Hypothesis 3a, the indirect effect of MA on math through WM and HCA reached a significance of β = −0.04, 95%CI [−0.105; −0.00]. On the contrary, the indirect effect via IC did not result statistically significant, with β = −0.03, 95%CI [−0.08; 0.00] (see Figure 1). The model also showed a direct effect of MA on math achievement (β = −0.27, *p* < .01), supporting Hypothesis 3b. Additional figures were included in the Supplementary Materials (Figures S1 and S2).

## 4. Discussion

The main aim of this study was to investigate how cognitive and emotional factors influence math in seventh graders, with an emphasis on WM, IC, HCA, and MA. Our preliminary analyses clearly show a positive relationship among our cognitive variables and a negative relationship with MA. In line with the literature, math showed a weak correlation with WM and IC, corroborating the idea that WM and IC may aid math and confirming our first Hypothesis 1. Specifically, WM may contribute to math achievement by retaining and elaborating information necessary for the resolution of math problems (Baddeley 2000; Peng et al. 2016), while IC may contribute by discarding and preventing the processing of irrelevant information (Bull and Lee 2014; Emslander and Scherer 2022). Further, a positive relationship emerged between WM and IC, suggesting that these two functions might be strictly and reciprocally related (Diamond 2013; Miyake and Friedman 2012). HCA showed a moderate correlation with math, which is consistent with a large body of evidence that found that these two factors are strongly related but still distinguishable (Deary et al. 2007; Giofrè et al. 2017). 

Our findings showed that WM, IC, and HCA are different and distinguishable constructs, and importantly, HCA were a substantial predictor of math achievement, even above other functions, such as WM and IC, confirming our Hypothesis 2 and aligning with previous research (Giofrè et al. 2017). Additionally, we partially confirmed Hypothesis 2a, as we found that HCA mediated the association between math achievement and WM, which is not surprising because WM seems more strongly related to math in younger children (Alloway and Alloway 2010; Giofrè et al. 2014), while in older children, intelligence plays a larger role (Demetriou et al. 2013). It is possible that WM alone is not sufficient to explain math achievement because the latter may require additional abilities, such as knowledge, thinking, and problem-solving. Likewise, while our first findings showed that math was moderately related to IC, subsequent analyses showed that this relationship was no longer significant. 

In regard to our Hypothesis 3, a negative correlation emerged between MA and math achievement, as these two factors were consistently negatively related (Ashcraft and Moore 2009; Caviola et al. 2022). Regarding our hypotheses, we found partial support for Hypothesis 3a, as our results showed that MA had a negative impact on WM, resulting in lower performance in math. This is consistent with prior research, suggesting that higher levels of MA might impair cognitive functions, such as WM, resulting in lower math performance (Ashcraft and Moore 2009). Our findings are partially consistent with the Attentional Control Theory developed by Eysenck et al. (2007). According to this theory, anxiety impairs cognitive abilities, such as attentional systems, thus resulting in an impairment in general performance (Eysenck et al. 2007). On the one hand, our results showed an indirect effect of MA on math achievement via WM and HCA. MA may have interfered with WM resources, thus leading to reduced efficiency and effectiveness in the math tasks. On the other hand, even though we found a significant negative relationship between MA and IC, we did not find an indirect effect of MA via IC on math achievement. This last result does not align with the Attentional Control Theory, which would expect negative effects to disrupt attentional resources while performing complex tasks. In other words, the WM and IC are strongly related constructs, so when both factors are included in the same model, the factor with the stronger effect (WM) tends to suppress the influence of the factor with the weaker effect, specifically IC. In relation to our study, math was assessed using a standardized battery without time constraints. As a result, participants may have spent more time on challenging items, which suggests that IC might play a less significant role in such tasks (Van den Bussche et al. 2020). 

Finally, our findings showed a direct effect of MA on math achievement, supporting Hypothesis 3b. This is not surprising, as, in fact, in line with this result, recent meta-analytic work has shown a consistent direct effect of MA, over and above WM, in influencing math achievement (Caviola et al. 2022). This last result raises an important question about the mechanisms that might drive this relationship. On the one hand, the direct association between MA and math achievement could be attributed to longitudinal changes that might occur in early schooling and then consolidate later, such as habitual avoidance and decreased involvement in math-related tasks (Sorvo et al. 2022; Suárez-Pellicioni et al. 2016). Since our sample consisted of middle school children, it is likely that these behaviors were already firmly in place by this stage. On the other hand, it is likely that other factors may play a mediating role in this relationship (Carey et al. 2016). In fact, research indicates that protective factors, such as academic self-esteem (Giofrè et al. 2017) and ego-resiliency (Donolato et al. 2020), can moderate the association between anxiety and math achievement. 

Some limits should be recognized. We only had a limited sample which may restrict the generalizability of the findings. Also, one potential disadvantage of our sample is the wide age range. In the Italian education system, it is possible to find students whose age does not align with the typical range for their grade, likely due to immigration-related school placement adjustments or individual educational needs. As a result, some children might not be the appropriate age for their grade level. While this scenario represents actual classroom composition, it might introduce variability, which should be considered when evaluating the results. Another potential problem is the use of only self-reported measures to assess MA. Furthermore, research suggests that diverse forms of anxiety might interfere with performance in math (Carey et al. 2017; Hill et al. 2016). Future research should focus on these aspects by combining all these measures into a single model and investigating both direct and indirect links between negative and positive emotions. Future research should strive to replicate these findings across different populations to improve external validity. Including more objective measurements of anxiety, such as physiological measures, may result in a more accurate assessment. Future research should also investigate the long-term effects of these factors. A longitudinal design would offer greater insights into how these characteristics develop over time and how early interventions could mitigate MA and improve math achievement.

Despite these limitations, the results of the present study have important implications for educational practice and intervention. Since HCA and WM play such an important role in predicting math achievement, cognitive training programs aiming at strengthening these abilities may result in better math education outcomes. Also, interventions that target EFs may also help students with complicated problem-solving tasks. Moreover, educators and psychologists should tackle MA and negative experiences in general. Because MA impairs cognitive functioning, interventions intended at lowering anxiety, such as mindfulness training or cognitive-behavioral approaches, may enhance both emotional well-being and math achievement.

## 5. Conclusions

This study emphasizes the importance of cognitive abilities, particularly HCA, and emotional elements, such as MA in influencing math. HCA mediated the effect of WM, whereas MA had an influence on performance, both directly and indirectly, through WM. These findings highlight the need for interventions to reduce anxiety in order to improve math outcomes.

## Figures and Tables

**Table 1:** Correlations (top) and descriptive statistics (bottom).

	1.	2.	3.	4.	5.
1. Math	-				
2. HCA	0.51	-			
3. IC	0.33	0.39	-		
4. WM	0.41	0.53	0.41	-	
5. MA	−0.38	−0.25	−0.34	−0.23	-
*M*	13.05	28.62	47.52	66.13	23.32
*SD*	5.67	10.59	14.08	12.82	6.18

**Table 2:** Path models.

Model	*χ* ^2^	*df*	*CFI*	*TLI*	*RMSEA*	*SRMR*	*AIC*	Δ*AIC*
Measurement model								
Model 0	75.94	68	0.97	0.96	0.04	0.07	6240.47	
Path analysis								
Model 1	0	0	1	1	<0.001	<0.001	2073.91	
Model 2	3.74	2	0.97	0.91	0.11	0.06	2073.66	−0.47
Model 3	2.19	2	1.00	0.99	0.04	0.03	2064.62	−9.04

Note. Model 0 is the measurement model; Model 1 is a “just-identified” model whose *df* are 0.

## Data Availability

The data presented in this study are available in OSF at https://osf.io/6hmds/?view_only=7983a67644c3469a9ee4637aae3a6081 (accessed on 1 March 2025).

## Supplementary Materials

The following supporting information can be downloaded at: https://www.mdpi.com/article/10.3390/jintelligence13040044/s1, Figure S1: Standardized solution for Model 1; Figure S2: Standardized solution for Model 2; Table S1: Correlations between mean age (in months) and each measure.

## References

[B1-jintelligence-13-00044] Agostino Alba, Johnson Janice, Pascual-Leone Juan (2010). Executive functions underlying multiplicative reasoning: Problem type matters. Journal of Experimental Child Psychology.

[B2-jintelligence-13-00044] Allen Katie, Giofrè David, Higgins Steve, Adams John (2020). Working memory predictors of mathematics across the middle primary school years. British Journal of Educational Psychology.

[B3-jintelligence-13-00044] Alloway Tracy Packiam, Alloway Ross G. (2010). Investigating the predictive roles of working memory and IQ in academic attainment. Journal of Experimental Child Psychology.

[B4-jintelligence-13-00044] Amoretti Guido, Bazzini Luciana, Pesci Angela, Reggiani Maria (1997). MAT-2. Test di Matematica.

[B5-jintelligence-13-00044] Andersson Ulf (2007). The Contribution of Working Memory to Children’s Mathematical Mathematical Word Problem Solving. Applied Cognitive Psychology.

[B6-jintelligence-13-00044] Andersson Ulf (2008). Working memory as a predictor of written arithmetical skills in children: The importance of central executive functions. British Journal of Educational Psychology.

[B7-jintelligence-13-00044] Ashcraft Mark H. (2002). Math anxiety: Personal, educational, and cognitive consequences. Current Directions in Psychological Science.

[B8-jintelligence-13-00044] Ashcraft Mark H., Moore Alex M. (2009). Mathematics anxiety and the affective drop in performance. Journal of Psychoeducational Assessment.

[B9-jintelligence-13-00044] Baddeley Alan (2000). The episodic buffer: A new component of working memory?. Trends in Cognitive Sciences.

[B10-jintelligence-13-00044] Beilock Sian L., Maloney Erin A. (2015). Math Anxiety: A Factor in Math Achievement Not to Be Ignored. Policy Insights from the Behavioral and Brain Sciences.

[B11-jintelligence-13-00044] Berg Derek H. (2008). Working memory and arithmetic calculation in children: The contributory roles of processing speed, short-term memory, and reading. Journal of Experimental Child Psychology.

[B12-jintelligence-13-00044] Bonato Mario, Fabbri Sara, Umiltà Carlo, Zorzi Marco (2007). The Mental Representation of Numerical Fractions: Real or Integer?. Journal of Experimental Psychology: Human Perception and Performance.

[B13-jintelligence-13-00044] Borella Erika, de Ribaupierre Anik (2014). The role of working memory, inhibition, and processing speed in text comprehension in children. Learning and Individual Differences.

[B14-jintelligence-13-00044] Borst Grégoire, Simon Grégory, Vidal Julie, Houdé Olivier (2013). Inhibitory control and visuo-spatial reversibility in piaget’s seminal number conservation task: A high-density ERP study. Frontiers in Human Neuroscience.

[B15-jintelligence-13-00044] Brookman-Byrne Annie, Mareschal Denis, Tolmie Andrew K., Dumontheil Iroise (2018). Inhibitory control and counterintuitive science and maths reasoning in adolescence. PLoS ONE.

[B16-jintelligence-13-00044] Bull Rebecca, Scerif Georgia (2001). Executive functioning as a predictor of children’s mathematics ability: Inhibition, switching, and working memory. Developmental Neuropsychology.

[B17-jintelligence-13-00044] Bull Rebecca, Lee Kerry (2014). Executive functioning and mathematics achievement. Child Development Perspectives.

[B18-jintelligence-13-00044] Burgoyne Alexander P., Tsukahara Jason S., Mashburn Cody A., Pak Richard, Engle Randall W. (2023). Nature and Measurement of Attention Control. Journal of Experimental Psychology: General.

[B19-jintelligence-13-00044] Burnham Kenneth, Anderson David (2004). Model Selection and Multimodel Inference. A Practical Information-Theoretic Approach.

[B20-jintelligence-13-00044] Campos Isabel S., Almeida Leandro S., Ferreira Aristides I., Martinez Luis F., Ramalho Glória (2013). Cognitive processes and math performance: A study with children at third grade of basic education. European Journal of Psychology of Education.

[B21-jintelligence-13-00044] Carey Emma, Devine Amy, Hill Francesca, Szucs Dénes (2017). Differentiating anxiety forms and their role in academic performance from primary to secondary school. PLoS ONE.

[B22-jintelligence-13-00044] Carey Emma, Hill Francesca, Devine Amy, Szücs Dénes (2016). The chicken or the egg? The direction of the relationship between mathematics anxiety and mathematics performance. Frontiers in Psychology.

[B23-jintelligence-13-00044] Caviola Sara, Primi Caterina, Chiesi Francesca, Mammarella Irene C. (2017). Psychometric properties of the Abbreviated Math Anxiety Scale (AMAS) in Italian primary school children. Learning and Individual Differences.

[B24-jintelligence-13-00044] Caviola Sara, Toffalini Enrico, Giofrè David, Ruiz Jessica Mercader, Szűcs Dénes, Mammarella Irene C. (2022). Math Performance and Academic Anxiety Forms, from Sociodemographic to Cognitive Aspects: A Meta-analysis on 906,311 Participants. Educational Psychology Review.

[B25-jintelligence-13-00044] Caviola Sara, Mammarella Irene C., Lucangeli Daniela, Cornoldi Cesare (2014). Working memory and domain-specific precursors predicting success in learning written subtraction problems. Learning and Individual Differences.

[B26-jintelligence-13-00044] Caviola Sara, Colling Lincoln J., Mammarella Irene C., Szűcs Dénes (2020). Predictors of mathematics in primary school: Magnitude comparison, verbal and spatial working memory measures. Developmental Science.

[B27-jintelligence-13-00044] Chen Edward H., Bailey Drew H. (2021). Dual-task studies of working memory and arithmetic performance: A meta-analysis. Journal of Experimental Psychology: Learning Memory and Cognition.

[B28-jintelligence-13-00044] Choe Kyoung Whan, Jenifer Jalisha B., Rozek Christopher S., Berman Marc G., Beilock Sian L. (2019). Calculated avoidance: Math anxiety predicts math avoidance in effort-based decision-making. Science Advances.

[B29-jintelligence-13-00044] Cragg Lucy, Gilmore Camilla (2014). Skills underlying mathematics: The role of executive function in the development of mathematics proficiency. Trends in Neuroscience and Education.

[B30-jintelligence-13-00044] Cragg Lucy, Keeble Sarah, Richardson Sophie, Roome Hannah E., Gilmore Camilla (2017). Direct and indirect influences of executive functions on mathematics achievement. Cognition.

[B31-jintelligence-13-00044] Daker Richard J., Gattas Sylvia U., Sokolowski H. Moriah, Green Adam E., Lyons Ian M. (2021). First-year students’ math anxiety predicts STEM avoidance and underperformance throughout university, independently of math ability. NPJ Science of Learning.

[B32-jintelligence-13-00044] Deary Ian J., Strand Steve, Smith Pauline, Fernandes Cres (2007). Intelligence and educational achievement. Intelligence.

[B33-jintelligence-13-00044] Demetriou Andreas, Spanoudis George, Rosén Monica, Hansen Kajsa Yang, Wolff Ulrika (2017). Mind and Intelligence: Integrating Developmental, Psychometric, and Cognitive Theories of Human Mind BT—Cognitive Abilities and Educational Outcomes: A Festschrift in Honour of Jan-Eric Gustafsson.

[B34-jintelligence-13-00044] Demetriou Andreas, Spanoudis George, Shayer Michael, Mouyi Antigoni, Kazi Smaragda, Platsidou Maria (2013). Cycles in speed-working memory-G relations: Towards a developmental-differential theory of the mind. Intelligence.

[B35-jintelligence-13-00044] Demetriou Andreas, Spanoudis George, Shayer Michael, Van der Ven Sanne, Brydges Christopher R., Kroesbergen Evelyn, Podjarny Gal, Swanson H. Lee (2014). Relations between speed, working memory, and intelligence from preschool to adulthood: Structural equation modeling of 14 studies. Intelligence.

[B36-jintelligence-13-00044] Diamond Adele (2013). Executive functions. Annual Review of Psychology.

[B37-jintelligence-13-00044] Distefano Christine, Zhu Min, Mindrila Diana (2008). Understanding and Using Factor Scores: Considerations for the Applied Researcher. Practical Assessment, Research, and Evaluation.

[B38-jintelligence-13-00044] Donolato Enrica, Toffalini Enrico, Giofrè David, Caviola Sara, Mammarella Irene C. (2020). Going Beyond Mathematics Anxiety in Primary and Middle School Students: The Role of Ego-Resiliency in Mathematics. Mind, Brain, and Education.

[B39-jintelligence-13-00044] Emslander Valentin, Scherer Ronny (2022). The Relation Between Executive Functions and Math Intelligence in Preschool Children: A Systematic Review and Meta-Analysis. Psychological Bulletin.

[B40-jintelligence-13-00044] Enders Craig K. (2020). Applied Missing Data Analysis.

[B41-jintelligence-13-00044] Eysenck Michael W., Derakshan Nazanin, Santos Rita, Calvo Manuel G. (2007). Anxiety and cognitive performance: Attentional control theory. Emotion.

[B42-jintelligence-13-00044] Foley Alana E., Herts Julianne B., Borgonovi Francesca, Guerriero Sonia, Levine Susan C., Beilock Sian L. (2017). The Math Anxiety-Performance Link: A Global Phenomenon. Current Directions in Psychological Science.

[B43-jintelligence-13-00044] Frey Meredith C., Detterman Douglas K. (2004). Scholastic Assessment or g?. Psychological Science.

[B44-jintelligence-13-00044] Friso-Van Den Bos Ilona, Van Der Ven Sanne H. G., Kroesbergen Evelyn H., Van Luit Johannes E.H. (2013). Working memory and mathematics in primary school children: A meta-analysis. Educational Research Review.

[B45-jintelligence-13-00044] Giofrè David, Borella Erika, Mammarella Irene Cristina (2017). The relationship between intelligence, working memory, academic self-esteem, and academic achievement. Journal of Cognitive Psychology.

[B46-jintelligence-13-00044] Giofrè David, Mammarella Irene C., Cornoldi Cesare (2013a). The structure of working memory and how it relates to intelligence in children. Intelligence.

[B47-jintelligence-13-00044] Giofrè David, Mammarella Irene C., Ronconi Lucia, Cornoldi Cesare (2013b). Visuospatial working memory in intuitive geometry, and in academic achievement in geometry. Learning and Individual Differences.

[B48-jintelligence-13-00044] Giofrè David, Mammarella Irene Cristina, Cornoldi Cesare (2014). The relationship among geometry, working memory, and intelligence in children. Journal of Experimental Child Psychology.

[B49-jintelligence-13-00044] Hill Francesca, Mammarella Irene C, Devine Amy, Caviola Sara, Passolunghi Maria Chiara, Szűcs Dénes (2016). Maths anxiety in primary and secondary school students: Gender differences, developmental changes and anxiety specificity. Learning & Individual Differences.

[B50-jintelligence-13-00044] Hornung Caroline, Schiltz Christine, Brunner Martin, Martin Romain (2014). Predicting first-grade mathematics achievement: The contributions of domain-general cognitive abilities, nonverbal number sense, and early number competence. Frontiers in Psychology.

[B51-jintelligence-13-00044] Hu Li Tze, Bentler Peter M. (1999). Cutoff criteria for fit indexes in covariance structure analysis: Conventional criteria versus new alternatives. Structural Equation Modeling.

[B52-jintelligence-13-00044] Jacob Robin, Parkinson Julia (2015). The Potential for School-Based Interventions That Target Executive Function to Improve Academic Achievement: A Review. Review of Educational Research.

[B53-jintelligence-13-00044] Johnson Heather Lynn (2015). Secondary Students’ Quantification of Ratio and Rate: A Framework for Reasoning about Change in Covarying Quantities. Mathematical Thinking and Learning.

[B54-jintelligence-13-00044] Johnson Wendy, Bouchard Thomas J. (2005). The structure of human intelligence: It is verbal, perceptual, and image rotation (VPR), not fluid and crystallized. Intelligence.

[B55-jintelligence-13-00044] Johnson Wendy, Nijenhuis Jan te, Bouchard Thomas J. (2008). Still just 1 g: Consistent results from five test batteries. Intelligence.

[B56-jintelligence-13-00044] Jorgensen Terrence D., Pornprasertmanit Sunthud, Schoemann Alexander M., Rosseel Yves (2022). semTools: Useful Tools for Structural Equation Modeling. https://cran.r-project.org/package=semTools.

[B57-jintelligence-13-00044] Kline Rex B. (2011). Principles and Practice of Structural Equation Modeling.

[B58-jintelligence-13-00044] Krajewski Kristin, Schneider Wolfgang (2009). Exploring the impact of phonological awareness, visual-spatial working memory, and preschool quantity-number competencies on mathematics achievement in elementary school: Findings from a 3-year longitudinal study. Journal of Experimental Child Psychology.

[B59-jintelligence-13-00044] Kroesbergen Evelyn H., Van Luit Johannes E. H., Van Lieshout Ernest C. D. M., Van Loosbroek Erik, Van de Rijt Bas A. M. (2009). Individual Differences in Early Numeracy. Journal of Psychoeducational Assessment.

[B60-jintelligence-13-00044] Lee Kerry, Lee Hon Wah (2019). Inhibition and Mathematical Performance: Poorly Correlated, Poorly Measured, or Poorly Matched?. Child Development Perspectives.

[B61-jintelligence-13-00044] Lee Kerry, Bull Rebecca, Ho Ringo M.H. (2013). Developmental changes in executive functioning. Child Development.

[B62-jintelligence-13-00044] Lee Kerry, Ng Swee Fong, Ng Ee Lynn, Lim Zee Ying (2004). Working memory and literacy as predictors of performance on algebraic word problems. Journal of Experimental Child Psychology.

[B63-jintelligence-13-00044] Lerner Matthew D., Lonigan Christopher J. (2014). Executive Function Among Preschool Children: Unitary Versus Distinct Abilities. Journal of Psychopathology and Behavioral Assessment.

[B64-jintelligence-13-00044] Li Jingguang, Zhao Yajun, Zhou Shan, Pu Yuling, He Hongyu, Zhao Ming (2020). Set-shifting ability is specifically linked to high-school science and math achievement in Chinese adolescents. PsyCh Journal.

[B65-jintelligence-13-00044] Lubin Amélie, Vidal Julie, Lanoë Céline, Houdé Olivier, Borst Grégoire (2013). Inhibitory control is needed for the resolution of arithmetic word problems: A developmental negative priming study. Journal of Educational Psychology.

[B66-jintelligence-13-00044] Miller Heather, Bichsel Jacqueline (2004). Anxiety, working memory, gender, and math performance. Personality and Individual Differences.

[B67-jintelligence-13-00044] Miller Michael R., Giesbrecht Gerald F., Müller Ulrich, McInerney Robert J., Kerns Kimberly A. (2012). A Latent Variable Approach to Determining the Structure of Executive Function in Preschool Children. Journal of Cognition and Development.

[B68-jintelligence-13-00044] Miyake Akira, Friedman Naomi P. (2012). The nature and organization of individual differences in executive functions: Four general conclusions. Current Directions in Psychological Science.

[B69-jintelligence-13-00044] Miyake Akira, Friedman Naomi P., Emerson Michael J., Witzki Alexander H., Howerter Amy, Wager Tor D. (2000). The Unity and Diversity of Executive Functions and Their Contributions to Complex “Frontal Lobe” Tasks: A Latent Variable Analysis. Cognitive Psychology.

[B70-jintelligence-13-00044] Navarro Jose I., Aguilar Manuel, Alcalde Concepcion, Ruiz Gonzalo, Marchena Esperanza, Menacho Inmaculada (2011). Inhibitory Processes, Working Memory, Phonological Awareness, Naming Speed, and Early Arithmetic Achievement. The Spanish Journal of Psychology.

[B71-jintelligence-13-00044] Nelwan Michel, Friso-van den Bos Ilona, Vissers Constance, Kroesbergen Evelyn (2022). The relation between working memory, number sense, and mathematics throughout primary education in children with and without mathematical difficulties. Child Neuropsychology.

[B72-jintelligence-13-00044] Nunes Terezinha, Bryant Peter, Evans Deborah, Barros Rossana (2015). Assessing Quantitative Reasoning in Young Children. Mathematical Thinking and Learning.

[B73-jintelligence-13-00044] Passolunghi Maria Chiara, Lanfranchi Silvia (2012). Domain-specific and domain-general precursors of mathematical achievement: A longitudinal study from kindergarten to first grade. British Journal of Educational Psychology.

[B74-jintelligence-13-00044] Paxton Pamela, Curran Patrick J., Bollen Kenneth A., Kirby Jim, Chen Feinian (2001). Monte Carlo Experiments: Design and Implementation. Structural Equation Modeling: A Multidisciplinary Journal.

[B75-jintelligence-13-00044] Peng Peng, Namkung Jessica, Barnes Marcia, Sun Congying (2016). A meta-analysis of mathematics and working memory: Moderating effects of working memory domain, type of mathematics skill, and sample characteristics. Journal of Educational Psychology.

[B76-jintelligence-13-00044] Pokropek Artur, Marks Gary N., Borgonovi Francesca (2022). How Much Do Students’ Scores in PISA Reflect General Intelligence and How Much Do They Reflect Specific Abilities?. Journal of Educational Psychology.

[B77-jintelligence-13-00044] Purpura David J., Baroody Arthur J., Lonigan Christopher J. (2013). The transition from informal to formal mathematical knowledge: Mediation by numeral knowledge. Journal of Educational Psychology.

[B78-jintelligence-13-00044] Purpura David J., Schmitt Sara A., Ganley Colleen M. (2017). Foundations of mathematics and literacy: The role of executive functioning components. Journal of Experimental Child Psychology.

[B79-jintelligence-13-00044] Qi Yue, Chen Yinghe, Yu Xiao, Yang Xiujie, He Xinyi, Ma Xiaoyu (2024). The relationships among working memory, inhibitory control, and mathematical skills in primary school children: Analogical reasoning matters. Cognitive Development.

[B80-jintelligence-13-00044] R Core Team (2024). R: A Language and Environment for Statistical Computing.

[B81-jintelligence-13-00044] Rivella Carlotta, Cornoldi Cesare, Caviola Sara, Giofrè David (2021). Learning a new geometric concept: The role of working memory and of domain-specific abilities. British Journal of Educational Psychology.

[B82-jintelligence-13-00044] Rochat Philippe (2023). The Evolution of Developmental Theories Since Piaget: A Metaview. Perspectives on Psychological Science.

[B83-jintelligence-13-00044] Rosseel Yves (2012). {lavaan}: An {R} Package for Structural Equation Modeling. Journal of Statistical Software.

[B84-jintelligence-13-00044] Roth Bettina, Becker Nicolas, Romeyke Sara, Schäfer Sarah, Domnick Florian, Spinath Frank M. (2015). Intelligence and school grades: A meta-analysis. Intelligence.

[B85-jintelligence-13-00044] RStudio Team (2024). RStudio: Integrated Development for R.

[B86-jintelligence-13-00044] Simanowski Stefanie, Krajewski Kristin (2019). Specific Preschool Executive Functions Predict Unique Aspects of Mathematics Development: A 3-Year Longitudinal Study. Child Development.

[B87-jintelligence-13-00044] Sorvo Riikka, Kiuru Noona, Koponen Tuire, Aro Tuija, Viholainen Helena, Ahonen Timo, Aro Mikko (2022). Longitudinal and situational associations between math anxiety and performance among early adolescents. Annals of the New York Academy of Sciences.

[B88-jintelligence-13-00044] Stavy Ruth, Tirosh Dina (2000). How Students (Mis-)Understand Science and Mathematics: Intuitive Rules.

[B89-jintelligence-13-00044] Suárez-Pellicioni Macarena, Núñez-Peña María Isabel, Colomé Àngels (2016). Math anxiety: A review of its cognitive consequences, psychophysiological correlates, and brain bases. Cognitive, Affective and Behavioral Neuroscience.

[B90-jintelligence-13-00044] Thurstone Louis L. (1937). The Primary Mental Abilities of Children. Journal of Educational Psychology.

[B91-jintelligence-13-00044] Tourva Anna, Spanoudis George (2020). Speed of processing, control of processing, working memory and crystallized and fluid intelligence: Evidence for a developmental cascade. Intelligence.

[B92-jintelligence-13-00044] Usai Maria Carmen, Viterbori Paola, Traverso Laura (2018). Preschool Executive Function Profiles: Implications for Math Achievement in Grades 1 and 3. Journal of Research in Childhood Education.

[B93-jintelligence-13-00044] Usai M. Carmen, Viterbori Paola, Traverso Laura, De Franchis Valentina (2014). Latent structure of executive function in five- and six-year-old children: A longitudinal study. European Journal of Developmental Psychology.

[B94-jintelligence-13-00044] Van den Bussche Eva, Vanmeert Katrien, Aben Bram, Sasanguie Dries (2020). Too anxious to control: The relation between math anxiety and inhibitory control processes. Scientific Reports.

[B95-jintelligence-13-00044] van Dijck Jean-Philippe, Fias Wim (2011). A working memory account for spatial-numerical associations. Cognition.

[B96-jintelligence-13-00044] Viterbori Paola, Traverso Laura, Usai M. Carmen (2017). The Role of Executive Function in Arithmetic Problem-Solving Processes: A Study of Third Graders. Journal of Cognition and Development.

[B97-jintelligence-13-00044] Wiebe Sandra A, Sheffield Tiffant, Nelson Jennifer Mize, Clark Caron A C, Chevalier Nicolas, Kimberly Andrews Espy (2010). The structure of executive function in 3-year-old children. Journal of Experimental Child Psychology.

[B98-jintelligence-13-00044] Wilkinson Hannah R., Smid Claudia, Morris Sarah, Farran Emily K., Dumontheil Iroise, Mayer Susan, Tolmie Andrew, Bell David, Porayska-Pomsta Katarzyna, Holmes Wayne (2020). Domain-Specific Inhibitory Control Training to Improve Children’s Learning of Counterintuitive Concepts in Mathematics and Science. Journal of Cognitive Enhancement.

[B99-jintelligence-13-00044] Yeniad Nihal, Malda Maike, Mesman Judi, Van Ijzendoorn Marinus H., Pieper Suzanne (2013). Shifting ability predicts math and reading performance in children: A meta-analytical study. Learning and Individual Differences.

[B100-jintelligence-13-00044] Zeidner Moshe (2007). Test Anxiety in Educational Contexts. Concepts, Findings, and Future Directions. Emotion in Education.

[B101-jintelligence-13-00044] Zhang Yuxin, Tolmie Andrew, Gordon Rebecca (2023). The Relationship between Working Memory and Arithmetic in primary school children: A meta-analysis. Brain Sciences.

