# Supplementary material for: Understanding the Role of Cognitive Abilities and Math Anxiety in Adolescent Math Achievement
Source: J Intell. 2025 Apr 3;13(4):44. doi: 10.3390/jintelligence13040044 (PMC12028032; doi:10.3390/jintelligence13040044)
Supplement: Supplementary file 1 [file jintelligence-13-00044-s001.zip › jintelligence-3496520-supplementary.pdf]

Supplementary Materials

Understanding the Role of Cognitive Abilities and Math Anxiety in Adolescent Math Achievement

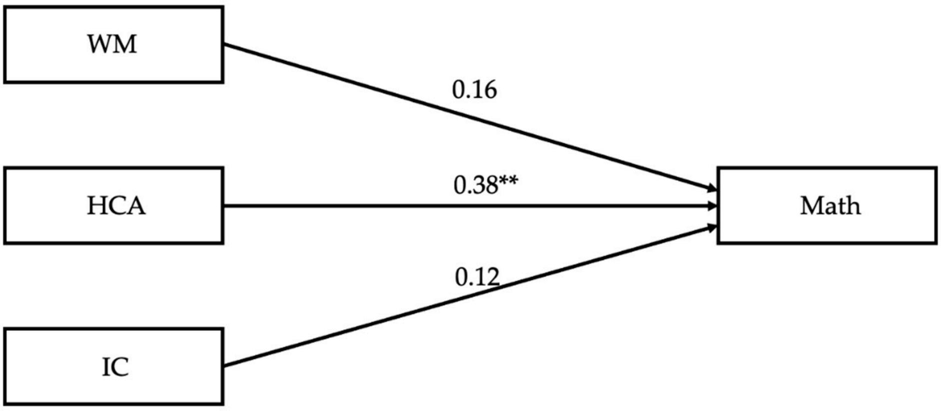

Figure S1. Standardized solution for Model 1.

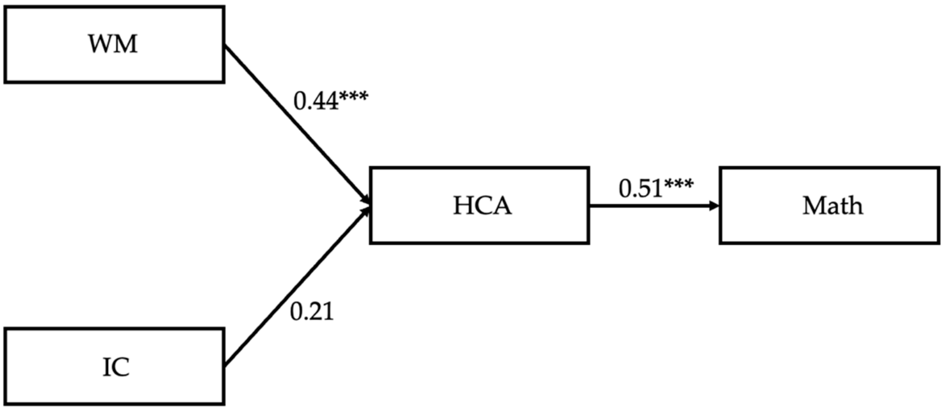

Figure S2. Standardized solution for Model 2.

Table S1. Correlations between mean age (in months) and each measure.

Test	*r*	*p*
Stroop_Score	−0.177	0.053
Flanker_Score	0.086	0.349
Simon_Score	−0.144	0.116
PMA_Ragionamento_TOT	−0.159	0.074
PMA_Verbale_TOT	−0.079	0.375
PMA_Spaziale_TOT	−0.044	0.622
AMAS_TOT	−0.016	0.860
Numeracy	−0.093	0.300
Geometry	−0.074	0.415
Statistics	−0.059	0.560
BackMatricesTot	−0.087	0.323
BackVerbalTot	−0.034	0.735
DualMatricesTot	0.036	0.686
DualVerbalTot	0.094	0.350
